# A Comparative Clinical Analysis of Esophagogastrostomy Versus Double-Tract Reconstruction Following Laparoscopic Proximal Gastrectomy

**DOI:** 10.14789/ejmj.JMJ25-0031-OA

**Published:** 2025-12-10

**Authors:** JUN CHEN, SUGURU YAMAUCHI, YUKINORI YUBE, YUJI ISHIBASHI, SATOSHI KANDA, HAJIME ORITA, SHINICHI OKA, SHINJI MINE, TETSU FUKUNAGA

**Affiliations:** 1 Department of Digestive and General Surgery, Juntendo University Urayasu Hospital, Chiba, Japan; 1 Department of Digestive and General Surgery, Juntendo University Urayasu Hospital, Chiba, Japan; 2 Department of Esophageal and Gastroenterological Surgery, Faculty of Medicine, Juntendo University, Tokyo, Japan; 2 Department of Esophageal and Gastroenterological Surgery, Faculty of Medicine, Juntendo University, Tokyo, Japan; 3 Department of Surgery, Johns Hopkins University School of Medicine, Baltimore, MD, USA; 3 Department of Surgery, Johns Hopkins University School of Medicine, Baltimore, MD, USA

**Keywords:** laparoscopic proximal gastrectomy, esophagogastrostomy, double-tract reconstruction, gastric cancer

## Abstract

**Introduction:**

Laparoscopic proximal gastrectomy (LPG) with esophagogastrostomy (EG) and double-tract reconstruction (DTR) for early gastric cancer of the upper stomach is a function-preserving surgery that retains oncological curative capability. However, direct comprehensive comparative analyses on the clinical outcomes of EG and DTR as reconstructive techniques following LPG are lacking. Therefore, this study investigated the short- and long-term clinical outcomes of EG and DTR following LPG.

**Materials and Methods:**

A retrospective comparative analysis was conducted based on a dataset compiled from two institutions. LPG cases for gastric cancer meeting eligibility criteria were divided into an EG group and a DTR group, and clinicopathological characteristics, perioperative outcomes, nutritional status, 3-year overall survival (OS), and cancer-specific survival (CSS) were analyzed. Furthermore, univariate and multivariate analyses were performed to identify risk factors for complications following LPG.

**Result:**

Among the 1198 patients, 104 received LPG, of whom 49 underwent EG and 41 underwent DTR. The DTR was significantly more frequently selected in patients with advanced cancer, while no significant differences were found in the other preoperative clinical factors. No significant differences were observed in perioperative outcomes, including postoperative complications, nutritional parameters, 3-year OS, and CSS. Multivariate analysis identified cardiac disease as a risk factor for postoperative complications following LPG.

**Conclusion:**

Short- and long-term clinical outcomes following LPG are equivalent between EG and DTR. For gastric cancer patients with cardiac disease undergoing LPG, careful attention is required for perioperative management.

## Introduction

Although the overall incidence and mortality rates of gastric cancer have been declining, the incidence of proximal gastric cancer (PGC) continues to rise globally, particularly in Western Europe and East Asia^1, 2)^. Laparoscopic proximal gastrectomy (LPG) has emerged as a function-preserving, minimally invasive surgical option for the management of PGC^3)^. Compared with laparoscopic total gastrectomy, LPG offers the potential advantage of preserving partial gastric reservoir capacity and physiological functions, with the aim of improving postoperative nutritional status and quality of life^3)^. According to the Japanese Gastric Cancer Treatment Guidelines, proximal gastrectomy is recommended for proximal tumors in which more than half of the distal stomach can be preserved, particularly for T1N0 tumors, considering appropriate surgical margins. In recent years, several studies have attempted to expand the application of proximal gastrectomy to patients with locally advanced gastric cancer located in the upper third of the stomach, provided that oncological cure remains feasible^4-6)^.

LPG presents particular challenges with respect to the choice of reconstruction methods, as no single technique has yet achieved universal acceptance or standardization. In Japan, the main reconstructive procedures performed after LPG are esophagogastrostomy (EG) and double-tract reconstruction (DTR)^6)^, each of which has its own advantages and disadvantages. EG is technically straightforward and preserves a more physiological food passage, but is frequently associated with a higher risk of postoperative reflux esophagitis and anastomotic stenosis^7, 8)^. In contrast, DTR has demonstrated superior efficacy in reducing the incidence of reflux by diverting a portion of the food stream through the jejunum, but it involves increased surgical complexity and is associated with extended operative times^9)^. Although the defining characteristics of each reconstruction method differ significantly, few direct and comprehensive comparative analyses have been conducted. In particular, evidence comparing these two techniques is limited for several key outcomes, such as the prevalence of specific postoperative complications, nutritional status, and long-term oncological results. The primary factor underlying this issue is considered to be largely attributable to the limited number of cases eligible for LPG procedures. While the number of upper gastric cancer cases indicated for LPG has been increasing in recent years, its adoption remains limited in clinical practice. According to the most recent 16th Nationwide Survey of Endoscopic Surgery in Japan, conducted by the Japanese Society of Endoscopic Surgery, only 1,220 out of 11,323 (10.8%) gastric cancer cases underwent LPG in 2021^10)^. This fact demonstrates the difficulty of designing and implementing large-scale case accumulation studies to optimize LPG technique. Particularly in Japan, surgery for gastric cancer is performed not only at university or high-volume hospitals but also at general hospitals, and surgical cases are not consolidated, which limits the number of LPG cases that each facility can experience. Furthermore, the choice of reconstructive technique for LPG may often be individualized based on tumor characteristics, anatomical features, eventually rely on the surgeon's expertise and facility-specific experience. The lack of consensus on the optimal reconstructive technique underscores the need for studies evaluating short and long-term perioperative outcomes and oncological safety between different reconstructive approaches.

Therefore, the aim of this study is to analyze real-world case selection, short- and long-term clinical outcomes of EG and DTR following LPG, and to propose a practical strategy of reconstruction after LPG for patients with upper gastric cancer. Additionally, we identify risk factors for complications following LPG.

## Materials and Methods

**Figure 1:**
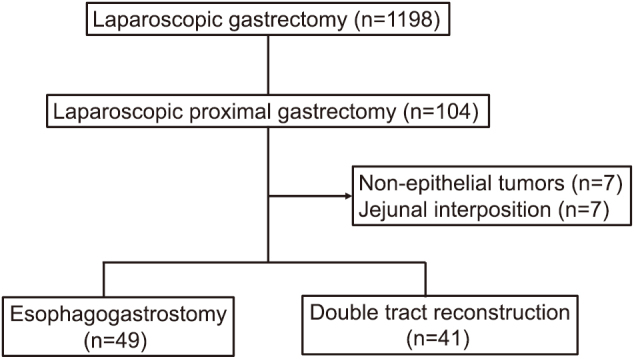
Study flow diagram Study design and patient grouping based on reconstruction methods following laparoscopic proximal gastrectomy. This flow diagram outlines the study design and patient classification. A total of 1198 patients with gastric cancer who underwent laparoscopic gastrectomy were retrospectively reviewed. After excluding ineligible cases, patients were divided into two groups according to reconstruction method.

From 1198 patients who underwent laparoscopic gastrectomy for gastric cancer at Juntendo University from 2016 to 2023 and Juntendo Urayasu Hospital from 2012 to 2023, 104 (8.7%) had received LPG (Figure 1). We performed a retrospective analysis by collecting clinical and pathological data on these 104 patients. These patients were confirmed for the following inclusion criteria, and those who met the exclusion criteria were excluded. Inclusion criteria were: (1) patients with confirmed gastric cancer of the upper stomach by preoperative pathological examination through upper gastrointestinal endoscopy, and (2) patients without distant metastasis before surgery. Exclusion criteria were: (1) patients with non-epithelial tumors, including gastrointestinal stromal tumors; (2) patients with jejunal interposition reconstruction following LPG. The indications for proximal gastrectomy and the extent of systematic lymphadenectomy are principally determined according to the gastric cancer treatment guidelines^6)^. The reconstruction method—either EG or DTR following LPG—was selected at the discretion of the operating surgeon or surgical team.

This retrospective observational study was approved by the Juntendo University Ethics Committee (E24-0393) and guaranteed patients the opportunity to refuse study participation by opting out (https://www.gcprec.juntendo.ac.jp/kenkyu/files/131420197867bec081be202.pdf).

### Date collection

Preoperative background was examined for sex, age, body mass index (BMI), comorbidity, including chronic obstructive pulmonary disease (COPD), cardiac disease, liver disease, diabetes mellitus, cerebrovascular disease, and other cancer history, American Society of Anesthesiologists physical status (ASA-PS), and neoadjuvant therapy. The diagnosis of gastric cancer is confirmed by preoperative upper gastrointestinal endoscopy, and preoperative tumor staging was performed by endoscopy and abdominopelvic computed tomography. The pathological diagnosis and classifications of gastric cancer were described according to the Japanese Classification of Gastric Carcinoma^6, 11)^. Perioperative analysis data included surgical time, intraoperative blood loss, number of lymph nodes dissected, postoperative hospitalization, and complications. Postoperative complications were those that occurred as either local or systemic complications within 90 days after surgery, and severity was classified according to the Clavien-Dindo (CD) classification system^12)^. The follow-up schedule for all gastric cancer patients was clinical examination and evaluation of clinical symptoms every 3 months, CT imaging at 6-month intervals, and upper gastrointestinal endoscopy at 1 year postoperatively unless recurrence was confirmed. The analysis of trends in nutritional status represented by body weight, hemoglobin, total protein, and albumin in this study, with percent reductions calculated based on preoperative values and one year postoperative values, respectively. Survival time, survival status (alive or deceased), and cause of death (cancer-specific or non-cancer-related) were collected for the analysis of overall survival (OS) and cancer-specific survival (CSS).

### Statistical analysis

Statistical analyses were performed using IBM SPSS Statistics for Mac, version 29.0.2.0 (IBM Corp., Armonk, N.Y., USA). All patients were grouped according to the surgical approach (EG or DTR) for subsequent analysis. The Shapiro-Wilk test was used to assess the normality of continuous variables. Normally distributed variables were reported as mean ± standard deviation, while non-normally distributed variables were reported as median [interquartile range (IQR)]. Levene's test was used to assess the homogeneity of variance, and Student t test and Welch t test were applied with the results. Non-normally distributed continuous variables were analyzed using the Mann-Whitney U test. Categorical and ordinal variables were presented as frequencies and percentages [n(%)] and analyzed with the chi-square test or Fisher’s exact test. All analyses reported the corresponding p values. Statistical significance was set at a two- sided p value of < 0.05. Propensity score matching was considered to adjust for possible confounders between the EG and DTR groups. However, the calculated propensity score was 0.21, indicating low overlap and poor feasibility for effective matching. Therefore, all comparative analyses in this study were performed using the original, unadjusted data sets. Group characteristics were compared using standardized statistical methods, and any variable with a significant imbalance was reported and considered in the interpretation of results.

Demographic and perioperative characteristics were included in the univariate analysis of all Clavien-Dindo graded complications, with odds ratios (ORs) and 95% confidence intervals (CIs) calculated and reported for each variable. ORs are calculated with the absence of comorbidity as the reference for the outcome. For categorical variables, the first group listed (e.g., EG for 'Reconstruction' and Male for 'Sex') is used as the reference group in the OR calculation. Statistically significant variables in univariate analysis (p < 0.05) were included in the multivariate analysis. Multivariate analysis identified independent risk factors for primary outcomes, and ORs, 95% CIs, and p values were reported.

## Results

### Clinicopathological characteristics

A total of 49 patients underwent EG and 41 underwent DTR during the study period (Table 1). There was no significant difference in sex distribution between the EG and DTR groups (p = 0.283). The median age was 71 (64-76) years in the EG group and 73 (65-82) years in the DTR group (p = 0.525). The mean BMI was 22.7 ± 3.0 in the EG group and 22.6 ± 2.9 in the DT group. No significant differences were observed between the two groups with respect to comorbidities, including COPD (p = 0.539), cardiac disease (p = 0.340), liver disease (p = 0.706), diabetes mellitus (p = 0.478), cerebrovascular disease (p = 0.241), and a history of other cancers (p = 0.374), as well as ASA-PS (p = 0.789). Histopathologically, adenocarcinoma was the most frequent diagnosis, followed by undifferentiated carcinoma; additionally, two cases of neuroendocrine tumor were included in the EG group. With respect to pathological findings, the T classification (p = 0.019), N classification (p = 0.013), and stage (p = 0.011) were all significantly higher in the DTR group. Tumor size was also significantly greater in the DTR group compared with the EG group, with median values of 38 (24-57) mm and 28 (20-40) mm, respectively. Neoadjuvant therapy was conducted in two patients in the EG group and one patient in the DTR group, with no significant difference between groups.

**Table 1:** Clinicopathological characteristics

	EG (n = 49)	DT (n = 41)	p value
Sex			0.283
Male	42	31	
Female	7	10	
Age	71 (64-76)	73 (65-82)	0.525
BMI (kg/m^2^)	22.7 ± 3.0	22.6 ± 2.9	0.948
Comorbidity			
COPD	4/49 (8.1%)	4/41 (9.7%)	0.539
Cardiac disease	6/49 (12.2%)	3/41 (7.3%)	0.34
Liver disease	1/49 (2.0%)	1/41 (2.4%)	0.706
Diabetes mellitus	13/49 (26.5%)	12/41 (29.2%)	0.478
Cerebrovascular disease	4/49 (8.1%)	1/41 (2.4%)	0.241
Other cancer history	5/49 (10.2%)	6/41 (14.6%)	0.374
ASA-PS (1/2/3)	6/35/8	5/27/9	0.789
Pathological diagnosis			0.258
Adenocarcinoma	42	39	
Undifferentiated cancer	5	2	
Neuroendocrine tumor	2	0	
pT (1/2/3/4)	32/9/5/3	21/2/11/7	0.019
pN (0/1/2/3)	41/7/1/0	25/6/7/3	0.013
pStage (1/2/3/4)	38/10/1/0	22/9/8/2	0.011
Tumor length diameter (mm)	28 (20-40)	38 (24-57)	0.03
Neoadjuvant therapy	2/49 (4.0%)	1/41 (2.4%)	1

EG, esophagogastrostomy; DTR, double-tract reconstruction; BMI, body mass index; COPD, chronic obstructive pulmonary disease; ASA-PS, American Society of Anesthesiologists physical status

### Surgical outcomes and postoperative complications

Surgical outcomes, including postoperative complications, are summarized in Table 2. The operative time was 330 (277-419) minutes in the EG group and 343 (280-413) minutes in the DTR group, with no significant difference between the two groups (p = 0.571). Intraoperative blood loss was 25 (14- 53) mL in the EG group and 20 (11-68) mL in the DTR group, showing no significant difference (p = 0.932). The number of dissected lymph nodes was 23 (19-32) in the EG group and 24 (18-33) in the DTR group (p = 0.942). Postoperative hospital stay was 11 (9-15) days in the EG group and 13 (10-16) days in the DTR group, without reaching statistical significance (p = 0.072).

Overall complications of any Clavien-Dindo (CD) grade occurred in 38.7% of patients in the EG group and 36.5% in the DTR group. When limited to complications of CD grade ≥ 2, the incidences were 18.3% and 21.9%, respectively, with no significant difference (p = 0.793). Anastomotic stricture occurred in one patient each (2.0% in EG, 2.4% in DTR). Anastomotic leakage was observed in three patients (6.1%) in the EG group and six patients (14.6%) in the DTR group, showing a higher tendency in the DTR group, although the difference was not statistically significant. The incidences of pancreatic fistula, intra-abdominal bleeding, internal hernia, and pulmonary infection did not differ significantly between the two groups.

**Table 2:** Surgical outcomes and postoperative complications

	EG (n = 49)	DT (n = 41)	p value
Surgical time (min)	330 (277-419)	343 (280-413)	0.571
Intraoperative blood loss (mL)	25 (14-53)	20 (11-68)	0.932
Dissected lymph nodes	23 (19-32)	24 (18-33)	0.942
Postoperative hospitalization (days)	11 (9-15)	13 (10-16)	0.072
All Complication	19/49 (38.7)	11/41 (36.5%)	1
CD≧2 Complication	9/49 (18.3%)	9/41 (21.9%)	0.793
Anastomotic complications	4/49 (8.1%)	7/41 (16.6%)	0.218
Anastomotic bleeding	0%	0%	
Anastomotic stricture	1/49 (2.0%)	1/41 (2.4%)	0.706
Anastomotic leakage	3/49 (6.1%)	6/41 (14.6%)	0.162
Pancreatic fistula	0/49 (0%)	1/41 (2.4%)	0.456
Intra-abdominal bleeding	0/49 (0%)	0/49 (0%)	
Intestinal obstruction	0/49 (0%)	1/41 (2.4%)	0.456
Internal hernia	0/49 (0%)	1/41 (2.4%)	0.456
Pulmonary infection	3/49 (6.1%)	3/41 (7.3%)	0.573

EG, esophagogastrostomy; DTR, double-tract reconstruction; CD, Clavien-Dindo

### Perioperative changes in body weight and nutritional status

**Figure 2:**
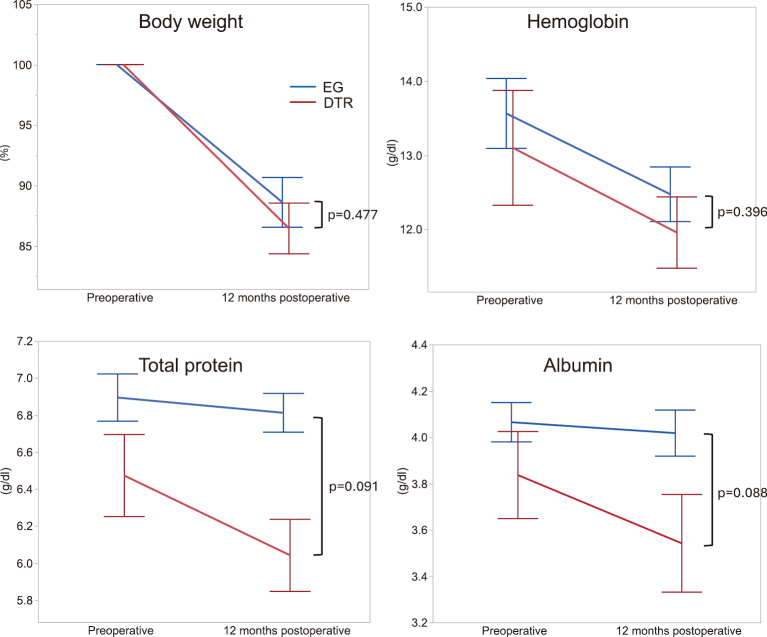
Postoperative nutrition indicator In this study, postoperative nutritional indicators included body weight, hemoglobin, total protein, and albumin. For trend analyses of nutritional status, the rates of decrease were calculated using both the preoperative values and the values at one year postoperatively as reference points, and comparative analyses were performed.

Perioperative changes in body weight and nutritional indices are shown in Figure 2. There were no significant differences in body weight or preoperative nutritional status between the EG and DTR groups. At one year postoperatively, the mean percentage body weight loss was 11.3% in the EG group and 13.5% in the DTR group, with no statistically significant difference (p = 0.477). Additionally, no significant differences were observed in hemoglobin, total protein, and albumin levels, as nutritional evaluation indices, between groups at one year after surgery (p = 0.396, p = 0.091, and p = 0.088, respectively).

### 3-year overall survival and cancer-specific survival

Long-term oncological outcomes are shown in Figure 3A and 3B. The 3-year survival rate was 95.9% in the EG group and 92.7% in the DTR group, with no significant difference (p = 0.919). The 3- year disease-free survival rate was 98% in the EG group and 95.1% in the DTR group, with no significant difference (p = 0.72).

**Figure 3A:**
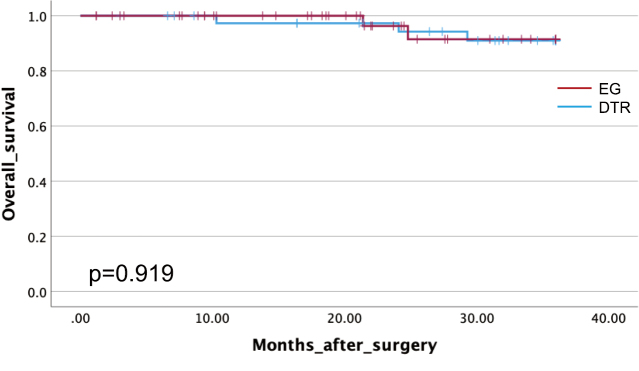
3-year overall survival Kaplan-Meier analysis comparing 3-year overall survival rates between patients who underwent esophagogastrostomy and double-tract reconstruction following LPG. No significant difference was observed between the groups (p = 0.919).

**Figure 3B:**
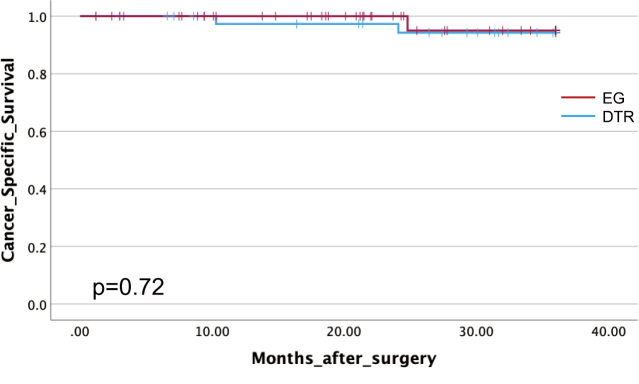
3-year cancer-specific survival Kaplan-Meier analysis comparing 3-year cancer-specific survival rates between esophagogastrostomy and double-tract reconstruction groups. No significant difference was noted between the techniques (p = 0.72).

### Univariate and multivariate risk analyses for all Clavien-Dindo graded complications

The results are presented in Table 3. Univariate analysis identified cardiac disease, coexistence of other cancers, and pathological T stage as independent risk factors for all Clavien-Dindo graded complications. Multivariate analysis revealed that cardiac disease remained an independent risk factor for all Clavien-Dindo graded complications. The method of reconstruction was not identified as a risk factor in either univariate or multivariate analyses.

**Table 3:** Univariate and multivariate risk analyses for all Clavien-Dindo graded complications

	Univariate		Multivariate	
	p value	OR (95%CI)	p value	OR (95%CI)
Reconstruction	0.831	0.91 (0.38-2.14)		
Sex	0.748	0.83 (0.28-2.46)		
Age	0.724	0.99 (0.95-1.03)		
BMI (kg/m^2^)	0.104	1.13 (0.97-1.31)		
COPD	0.986	0.98 (0.22-4.42)		
Cardiac disease	0.009	16.9 (2.01-142.48)	0.036	10.65 (1.16-97.31)
Liver disease	0.999	0		
Diabetes mellitus	0.484	0.70 (0.26-1.87)		
Cerebrovascular disease	0.916	1.10 (0.17-6.96)		
Other cancer	0.018	5.43 (1.33-22.20)	0.057	4.26 (0.96-18.91)
ASA-PS	0.069	2.13 (0.94-4.83)		
Pathological diagnosis	0.253	0.43 (0.10-1.81)		
pT	0.04	1.51 (1.01-2.25)	0.265	1.27 (0.83-1.97)
pN	0.076	1.64 (0.94-2.83)		
pStage	0.109	1.58 (0.90-2.76)		
Tumor length diameter (mm)	0.777	0.99 (0.98-1.01)		
Neoadjuvant therapy	0.872	0.81 (0.07-9.38)		
Surgical time (min)	0.248	1.00 (0.99-1.00)		
Intraoperative blood loss (mL)	0.492	1.00 (0.99-1.00)		
Dissected lymph node	0.654	0.99 (0.96-1.02)		

BMI, body mass index; COPD, chronic obstructive pulmonary disease; ASA, American Society of Anesthesiologists

## Discussion

Although DTR tended to be selected for more advanced cancer cases, there were no significant differences in perioperative outcomes, postoperative complications, weight loss, or postoperative nutritional status compared to EG, and long-term prognosis was also similar. Moreover, concomitant cardiac disease was identified as a significant risk factor for all Clavien-Dindo graded complications in the context of LPG, underscoring the importance of stringent perioperative management.

The tendency for DTR to be preferred in more advanced cases may be explained by its technical characteristics; DTR allows reconstruction regardless of residual stomach size following upper gastric resection and remnant esophageal length. Therefore, factors such as tumor location, size, and regional lymph node involvement likely influenced selection towards DTR. The report by Ikeda et al. described a large retrospective study using the Postgastrectomy Syndrome Assessment Scale sub-analysis^13)^. The study indicated that DTR was more likely to be performed in cases with smaller residual stomach and shorter esophagogastric anastomosis-diaphragm distances, which supported our finding. The equivalent oncological outcomes of EG and DTR in this cohort suggest that DTR is an appropriate alternative for cases where EG is not feasible. For instance, alternative approaches may be acceptable in situations where limited residual esophageal length or unexpected reduction of residual gastric capacity is encountered intraoperatively. Shifting to DTR from EG reconstruction during surgery, when warranted by anatomical or oncological considerations, does not appear to negatively affect patient prognosis and may be regarded as a clinically permissible strategy.

The latest systematic reviews indicate that, regarding perioperative outcomes, DTR tends to result in longer operative times, while EG is associated with a higher incidence of anastomotic stricture^14)^. Whereas, blood loss, anastomotic leakage, postoperative hospital stay, and nutritional indices showed no significant differences between methods^14)^. Our findings were nearly consistent with these reports, though operative times were comparable, and there were no significant differences in anastomotic- related complications. Surgical outcomes can be significantly influenced by subtle variations in technique between institutions and surgeons. Our analysis included cases from university hospitals and their directly affiliated hospitals, involving relatively standardized surgical teams and surgeons. This high degree of consistency in technique likely contributed to the stability of our perioperative results.

Pre-existing cardiac disease is a recognized risk factor for postoperative complications following laparoscopic gastrectomy^15-17)^, and associations with anastomotic leakage^15)^ and systemic complications^17)^ have been reported. The use of laparoscopy is known to affect numerous hemodynamic factors, including decreased cardiac index and stroke work index, as well as increased systemic and pulmonary vascular resistance^18, 19)^. The physiological changes induced by the establishment of intra-abdominal pneumoperitoneum have also been suggested to have the potential to cause chronic heart failure^20)^. Additionally, elderly patients, especially those with chronic heart failure or ischemic heart disease, demonstrate reduced physiological reserve to tolerate the hemodynamic alterations associated with laparoscopic surgery. Multiple cohort studies indicate that this diminished compensatory ability leads to a higher incidence of postoperative complications in this population^21-23)^. Due to limited available data, we were unable to analyze details of cardiac disease or its association with specific complications. However, the increased prevalence of cardiac complications in elderly gastric cancer patients undergoing laparoscopic gastrectomy highlights the need for heightened attention to these factors during the perioperative period. There have been no breakthrough discoveries that have fundamentally altered perioperative management strategies for patients with cardiac disease; however, meticulous implementation of classical care protocols remains essential. Comprehensive preoperative cardiac assessment, incorporating disease subtype, severity, and control status, as well as careful management of cardiac medications, is central to effective preoperative management^24)^. Intraoperatively, stabilization of hemodynamics, minimizing intra-abdominal pressure to the lowest effective level, optimal patient positioning, and appropriate ventilator management are pivotal in reducing cardiac workload during surgery^25)^. Additionally, strict adherence to preventive measures against postoperative thromboembolic events—which are closely related to underlying cardiac disease—remains imperative^26)^. The most recent European Association for Endoscopic Surgery and Society of American Gastrointestinal and Endoscopic Surgeons evidence-based recommendations for perioperative management emphasize the implementation of preoperative rehabilitation and enhancement of Enhanced Recovery After Surgery (ERAS) protocols as primary strategies to reduce postoperative complications in upper gastrointestinal surgery^27)^. Currently, there are no dedicated programs specifically tailored for patients with cardiac comorbidities; however, future research is warranted to develop individualized perioperative management approaches for this high-risk population.

Current guidelines recommend LPG only for Stage I early gastric cancer, resulting in very limited research on long-term oncological outcomes based on differences in reconstruction methods following LPG. This study, by employing a non-matched, real-world patient cohort, accurately captures the contemporary clinical tendency to select DTR for more advanced proximal gastric cancer cases. Our findings on the similarity in long-term outcomes between EG and DTR reconstruction, derived from our dataset including heterogeneous Stage II or higher advanced gastric cancers, may provide evidence for the applicability, safety, and feasibility of our surgical strategy. Our multicenter retrospective study may provide fundamental insights that promote further research in diverse areas such as oncologic outcomes, complications, postgastrectomy syndromes, and quality of life, particularly in studies of reconstruction following LPG for which large-scale prospective study designs are challenging.

## Limitation

This study was conducted as a retrospective observational analysis at a limited number of institutions with a relatively small sample size. The small sample size in this study may limit the statistical power to detect differences, particularly in uncommon complications. Thus, the possibility of type II errors cannot be excluded. There were significant differences in clinicopathological background, particularly as the DTR group included more advanced cases. Propensity score matching was considered to reduce intergroup bias, but was not feasible due to insufficient overlap. Therefore, results should be interpreted with caution as unadjusted comparisons have inherent limitations. The selection criteria between EG and DTR for each patient could not be confirmed from the datasets, potentially contributing to statistical bias. Furthermore, the potential influence of surgeon expertise on surgical outcomes could not be assessed due to unavailable of detailed data on operator experience between groups. Therefore, the findings may not fully represent substantial comparative outcomes. Validation of these results will require a prospective study employing a standardized protocol with sufficiently large sample sizes. Data constraints precluded a detailed analysis of cardiac disease subtypes or the association between specific complications and cardiac conditions, despite cardiac disease being identified as an independent risk factor for complications following LPG. Functional assessments regarding post-gastrectomy syndrome or postoperative quality of life were also not included in the present analysis. For further clarification of LPG as a function-preserving procedure, evaluation of postoperative complaints and quality of life may yield more meaningful insights. Both EG and DTR comprise a variety of technical subtypes, but, in order to maximize sample size and account for historical changes in procedural selection, these subtype methods were not analyzed separately in this study.

## Conclusion

In comparing EG and DTR for reconstruction after LPG, DTR was preferred for more advanced upper gastric cancer, with equivalent short- and long-term clinical outcomes. Cardiac disease is an independent risk factor for complications after LPG, requiring alertness in perioperative management.

## Author contributions

JC was responsible for data collection, statistical analysis, and manuscript drafting. SY, YY, YI, SK, and HO supported data interpretation and provided critical revisions to the manuscript. SO, SM, and TF provided supervisory oversight and comprehensive manuscript refinement. All authors participated in the preparation of the manuscript and gave their final approval for publication.

## Conflicts of interest statement

The authors declare that there are no conflicts of interest.
